# Differences in estimation of creatinine generation between renal function estimating equations in an Indian population: cross-sectional data from the Hyderabad arm of the Indian migration study

**DOI:** 10.1186/1471-2369-14-30

**Published:** 2013-02-04

**Authors:** Phillippa K Bailey, Charles RV Tomson, Sanjay Kinra, Shah Ebrahim, KV Radhakrishna, Hannah Kuper, Dorothea Nitsch, Yoav Ben-Shlomo

**Affiliations:** 1 The Richard Bright Renal Unit, Southmead Hospital, Westbury-on-Trym, Bristol BS10 5NB, UK; 2 London School of Hygiene and Tropical Medicine, Keppel Street, London, WC1E 7HT, UK; 3 National Institute of Nutrition, Hyderabad, India; 4 School of Social and Community Medicine, University of Bristol, Canynge Hall, 39 Whatley Road, Bristol, BS8 2PS, UK

**Keywords:** Creatinine, Ethnicity, Muscle mass, Renal function

## Abstract

**Background:**

Creatinine based formulae for estimating renal function developed in white populations may be less valid in other ethnic groups. We assessed the performance of various estimating formulae in an Indian population.

**Methods:**

917 subjects were recruited from the Hyderabad arm of the Indian Migration Study. Data were collected on comorbidity, serum creatinine and body composition from DXA scans. Renal function was compared using the modified Cockcroft-Gault, MDRD and CKD-EPI formulae. 24-hour creatinine production was derived from each estimate and the agreement with measured muscle mass examined. 24-hour creatinine production estimates were compared to that derived from a formula by Rule incorporating DXA measured muscle mass. Potential systematic biases were examined by age and eGFR. We assessed the association of renal function by each formula with hypertension and self-reported measures of vascular disease.

**Results:**

Mean modified Cockcroft-Gault eCCl was 98.8 ml/min/1.73 m^2^, MDRD eGFR 91.2 ml/min/1.73 m^2^ and CKD-EPI eGFR 96.3 ml/min/1.73 m^2^. MDRD derived 24-hour creatinine production showed the least age-related underestimation compared to the Rule formula. CKD-EPI showed a marked bias at higher eGFRs. All formulae showed similar strength associations with vascular disease and hypertension.

**Conclusions:**

Our analyses support the use of MDRD for estimating renal function in Indian populations. Further work is required to assess the predictive value of formulae for incident disease and complications of CKD.

## Background

Chronic kidney disease (CKD) is increasingly recognised as a public health problem, associated with an increased risk of cardiovascular disease, End-Stage Renal Failure (ESRF) requiring renal replacement therapy (RRT) and a reduced life expectancy [1]. In people with CKD, cardiovascular disease is the leading cause of death, and CKD is an independent risk factor for coronary heart disease (CHD) [2]. In the UK, crude relative acceptance rates for RRT in South Asians (from India, Pakistan and Bangladesh) is 3.5 times that seen in Whites [3]. The burden of CKD in India is not quantified but given the burden of diabetes, hypertension and cardiovascular disease, it is predicted to be high [4]. Information on CKD is needed to enable intervention at early stages of disease to prevent complications and progression to ESRF.

All creatinine-based formulae used to estimate renal function imply an estimation of creatinine generation rate based on demographic variables. It is recognised that methods of estimating Creatinine Clearance (CCl) and Glomerular Filtration Rate (GFR) developed in White populations may require adjustment for use in other ethnic groups [5,6]. African-American individuals have a higher generation of creatinine from muscle than White subjects, due mostly to greater skeletal muscle mass [7,8]. Thus co-efficients for African-Americans within MDRD and CKD-EPI result in higher values for estimated GFR (eGFR) than Whites for the same plasma creatinine concentration, age and gender [9]. Similar differences in skeletal muscle mass and creatinine generation exist between Indian and White populations [10]. To date, none of the renal function estimating formulae has been well validated in an Indian population. The CKD-EPI research group carried out work on developing an ‘Asian’ coefficient but this aimed to incorporate primarily Chinese and Japanese populations [5].

Rule and colleagues recently investigated the use of Dual-energy X-ray absorptiometry (DXA) measurements of muscle mass to estimate Urinary Creatinine Clearance (UCCl) and compared this to the use of demographic variables [11]. A model based on muscle mass and age had the best fit.

In this mixed urban-rural Indian population from Hyderabad we investigated different methods of estimating renal function, including Rule’s formula using measured muscle mass. The objective of this study was to validate the estimation of creatinine generation between different equations used to estimate GFR or CCl against DXA measured muscle mass in an Indian population. We derived 24-hour (24 h) creatinine production from each renal function estimate and compared this to measured muscle mass. This comparison provides a unique insight into the ability of demographic variables within each formula to estimate creatinine generation in an Indian population. Demographic variables in creatinine-based estimating formulae primarily account for differences in muscle mass, from which creatinine is largely derived.

We compared estimates of excretory renal function, and consequent classification of CKD, by different formulae. Further, we assessed the association of self-reported measures of vascular disease and hypertension with each estimate.

## Methods

### Study methodology

Using the framework of a cardiovascular risk factor screen study conducted in factories in north, central and south India [12] a sib-pair comparison study was designed. Details of this are reported elsewhere [13]. The original study was in four factories: Lucknow, Hindustan Aeronautics Ltd; Nagpur, Indorama Synthetics Ltd; Hyderabad, Bharat Heavy Electricals Ltd; and Bangalore, Hindustan Machine Tools Ltd. Factory workers and their co-resident spouses were recruited if they were rural-urban migrants using employer records as the sampling frame. Each migrant worker and spouse was asked to invite one non-migrant same-sex full-sibling, closest to them in age, residing in their rural place of origin. A 25% random sample of non-migrants was invited to participate in the study. Non-migrants were also asked to invite a sibling who resided in the same city but didn’t work in the factory. Information sheets were translated into local languages and signed (thumb print acceptable) and informed consent obtained. Ethics approval was from the All India Institute of Medical Sciences Ethics Committee, reference no. A-60/4/8/2004. Field work ran from March 2005 to December 2007. The Hyderabad arm of the Indian Migration Study is a follow-up study of the Hyderabad recruited subjects. The participants were invited to attend a screening clinic at the National Institute of Nutrition between January 2009 and December 2010.

### Clinic measures

Participants were interviewed and data collected on demographic factors, including socioeconomic status using a subset of 12 questions from the Standard of Living Index (SLI), a household level asset-based scale devised for Indian surveys [14]. These comprised house type, house ownership, toilet facility, lighting source, drinking water source, car/tractor, scooter, telephone, refrigerator, television, bicycle, radio, clock/watch, and weighted to give a maximum score of 33. Weighting was developed by the International Institute of Population Sciences. Individuals were classed as having ‘low’ (0-7), ‘middle’ (8-12) or ‘high’ (13-33) standards of living. Smoking was assessed as positive if individuals actively smoked or chewed tobacco. Few individuals reported past but not active smoking. A diagnosis of CHD and Stroke was made by self-report of a doctor diagnosis. Weight was measured twice without shoes using digital Seca scales (http://www.seca.com). Standing height was measured twice without shoes using a portable stadiometer (Leicester height measure; Chasmors Ltd, Camden, London, UK). Body Mass Index was calculated as weight(kg)/height(m)^2^. We used a validated oscillometric device (OMRON M5-I; Omron, Matsusaka Co, Japan) to measure blood pressure (BP) in the sitting position with appropriate cuff sizes. We took three measures 2-3 minutes apart, and averaged the last two measures for analyses. A diagnosis of hypertension was made if average systolic BP was ≥140 mmHg, average diastolic BP ≥90 mmHg or if there was report of a doctor diagnosis of hypertension [15].

Participants were asked to attend fasting and the time of their last meal was recorded. Creatinine analysis was performed using the rate-blanked compensated Jaffe method on a Roche COBAS-C311 autoanalyzer. The calibraton for this assay is traceable to isotope dilution mass spectrometry (IDMS). The Cardiac Biochemistry Lab, AIIMS, is part of the UK National External Quality Assessment Scheme (http://www.ukneqas.org.uk) to quality assure assays.

Whole body DXA scans were performed on a Hologic-DXA machine Discovery-A model (91% scans) or a Hologic-QDR-4500-Elite machine (http://www.hologic.com) (9% scans). Scans were individually checked for major artefacts and if present were removed from the analyses. As quality assurance, a spine phantom was scanned daily to check for acceptable ranges. Total skeletal muscle mass was determined from the lean body mass of all four extremities multiplied by 1.33 [16]. Body Surface Area was calculated by the Mosteller equation [17]. Renal function was estimated using four measures, including Rule’s formula incorporating measured muscle mass [11]:

Renal function estimating equations:

1. Creatinine Clearance by Cockcroft-Gault modified for Body Surface Area (BSA) by the Mosteller equation.

$$\begin{array}{l} \;\text{eCCl}=\left(140–\text{Age}\right)\;\times \;\text{Mass}\left(\text{kg}\right)\\ \;\times \;\left[0.85\;\mathrm{if}\;\text{female}\right]/\text{serum creatinine (mg/dl)}\\ \;=\text{ml}/\text{min} \end{array}$$

Serum creatinine (mg/dl)

$$\begin{array}{l} \text{Modified}\;\text{for}\;\text{BSA}=\text{eCCl}\;\times 1.73/\text{BSA}\\ \;=\text{ml}/\text{min}/1.73m^{2} \end{array}$$

BSA

2. The IDMS standardized Modification of Diet in Renal Disease (MDRD) formula

$$\begin{array}{l} \text{eGFR}=175\;\times (\;\text{Serum}\;\text{creatinine}\;{\left(\text{mg}/\text{dl}\right)}^{-1.154}\times \;\text{Ag}e^{-0.203}\\ \;\times \;\left[0.742\;\text{if}\;\text{female}\right]=\text{ml}/\text{min}/1.73m^{2} \end{array}$$

3. The CKD-EPI formulae

For ‘non-black females’:

If Serum creatinine ≤ 0.7 mg/dl

$$\begin{array}{l} \text{eGFR}=144\;\times \;{\left(\text{Serum}\;\text{creatinine}/0.7\right)}^{-0.329}\times \;0.993^{\text{Age}}\\ \;=\text{ml}/\text{min}/1.73m^{2} \end{array}$$

If Serum creatinine > 0.7 mg/dl

$$\begin{array}{l} \text{eGFR}=144\;\times \;{\left(\text{Serum}\;\text{creatinine}/0.7\right)}^{-1.209}\times \;0.993^{\text{Age}}\\ \;=\text{ml}/\text{min}/1.73m^{2} \end{array}$$

For ‘non-black males’:

If Serum creatinine ≤0.9 mg/dl

$$\begin{array}{l} \text{eGFR}=141\;\times \;{\left(\text{Serum}\;\text{creatinine}/0.9\right)}^{-0.411}\times \;0.993^{\text{Age}}\\ \;=\text{ml}/\text{min}/1.73m^{2} \end{array}$$

If Serum creatinine >0.9 mg/dl

$$\begin{array}{l} \text{eGFR}=141\;\times \;{\left(\text{Serum}\;\text{creatinine}/0.9\right)}^{-1.209}\times \;0.993^{\text{Age}}\\ \;=\text{ml}/\text{min}/1.73m^{2} \end{array}$$

4. Estimated creatinine clearance based on Rule formula DXA measured skeletal muscle mass and age

If age > 55 years:

$$\frac{\begin{array}{ccc} \text{eCrClagemuscle} & = & \text{exp}\;\left[6.37+0.029\;\times \;\left(\text{muscle}\;\text{in}\;\text{kg}/1.73m^{2}\right)\right)\\ & & \left(-\left[0.0071\;\times \;\text{age}-55\right]\right]=\text{ml}/\text{min}/1.73m^{2} \end{array}}{\left(\text{Serum}\;\text{creatinine}\;\left(\text{mg}/\text{dl}\right)\;\times \;14.4\right)}$$

If age ≤55 years:

$$\frac{\begin{array}{ccc} \text{eCrClagemuscle} & = & \exp\;[6.37+0.029]\\ & & \;\times \;\left(\text{muscle}\;\text{in}\;\text{kg}/1.73m^{2}\right)=\text{ml}/\text{min}/1.73m^{2} \end{array}}{\left(\text{Serum}\;\text{creatinine}\;\left(\text{mg}/\text{dl}\right)\;\times \;14.4\right)}$$

CKD was classified according to the KDOQI guidelines [18]. As information on proteinuria was unavailable, CKD 1 and 2 were not identified. We defined an eGFR <60 ml/min/1.73 m^2^ (CKD stages 3-5) as ‘CKD’ and an eGFR ≥60 ml/min/1.73 m^2^ as ‘No CKD’. In clinical practice, the diagnosis of CKD requires evidence of reduced function for ≥3 months; in epidemiological studies a single estimate of GFR is accepted [19].

All creatinine-based GFR formulae use the serum creatinine concentration and demographic variables to empirically derive an estimate of GFR. Serum creatinine concentration is determined by the arithmetical relationship between creatinine generation rate and glomerular filtration rate, whilst noting that CCl is not precisely the same as GFR, particularly when GFR is low, because of tubular secretion. Therefore the formulae are, in effect, different ways of estimating creatinine generation rate from demographic characteristics, estimating GFR from this together with the serum creatinine concentration. Creatinine generation rate should then be:

Creatinine generation rate (mmol/24 h) = CCl or GFR (ml/min) × serum creatinine (mmol/ml) × 1440 (min)

Given that creatinine generation rate is proportional to muscle mass, one would expect to find a linear correlation between the two. The tightness of this correlation would be a measure of the accuracy of each formula.

### Statistical analyses

Linear regression and Pearson’s correlation coefficient were used to investigate the relationship between each estimate of renal function and DXA measured skeletal muscle mass. Bland-Altman plots, which plot the difference (delta) between two formulae against the average, were used to visually compare the relative performance of each formula. To assess how each formula may perform across a range of ages, we regressed the difference in 24 h creatinine production as estimated by the DXA based Rule formula and the traditional formulae, against age to examine for any systematic bias (increasing/decreasing differences with age). We tested for any differences in the regression coefficients using a heterogeneity test.

All creatinine-based renal function formulae underperform systematically at high GFRs. Renal function estimates therefore underwent log_2_-transformation to allow this error to be more normally distributed. Therefore, one unit increase change is equivalent to doubling renal function. We used logistic regression to assess the association between the transformed renal function estimates and our disease end-points, vascular disease and hypertension, both unadjusted and adjusted for age. We undertook sex-stratified and combined analyses after formally testing for any sex interaction.

The study included related individuals who are not truly independent, so we used robust standard errors in our regression models to allow for any family clustering effect. This provides wider 95% confidence intervals than conventional methods. Statistical analyses were performed using Stata 11.

## Results

The study sample comprised 917 adult subjects (mean age 48.5 ± SD 8.3 years) and roughly equal numbers of men and women (Table 1). The crude mean muscle mass in women was 7.8 kg (95% CI 7.7-7.9) less than that in men. However, the BMI of women was 2.2 kg/m^2^ higher than that of men (95% CI 2.1-2.3). The mean eCCl by mCG was 98.8 ml/min/1.73 m^2^ and by Rule’s formula 103.2 ml/min/1.73 m^2^. eGFR as estimated by MDRD was 91.2 ml/min/1.73 m^2^ and by CKD-EPI 96.3 ml/min/1.73 m^2^. Generally, estimates of excretory renal function were higher in women than in men. In men the Rule formula generated the highest estimate of renal function, followed by CKD-EPI, mCG and MDRD. In women, the findings were slightly different with mCG generating a higher estimate than CKD-EPI.

**Table 1:** Baseline characteristics of Hyderabad arm of the Indian Migration Study stratified by sex

	**All**	**Male**	**Female**
No of observations	917	484 (52.8%)	433 (47.2%)
		**Mean (± S.D)**	
Age (years)	48.5 (±8.3)	50.2 (±8.4)	46.6 (±7.7)
Weight (kg)	65.4 (±11.9)	67.7 (±11.5)	62.8 (±11.9)
Height (cm)	159.0 (±8.7)	165.1 (±6.3)	152.3 (±5.6)
BMI (kg/m^2^)	25.9 (±4.4)	24.8 (±3.7)	27.0 (±4.8)
BSA (m^2^)	1.69 (±0.18)	1.76 (±0.17)	1.62 (±0.17)
Muscle Mass (kg)	24.1 (±5.4)	27.7 (±4.0)	19.9 (±3.4)
Muscle mass (kg per 1.73 m^2^)	24.4 (±3.7)	27.2 (±2.1)	21.1 (±1.9)
Systolic Blood Pressure (mmHg)	122 (±16)	125 (±16)	117 (±15)
Diastolic Blood Pressure (mmHg)	81(±10)	82 (±10)	79 (±9)
Socio-economic Status – Low	16 (1.7%)	5 (1.0%)	11 (2.5%)
Socio-economic Status – Middle	44 (4.8%)	25 (5.2%)	19 (4.4%)
Socio-economic Status – High	857 (93.5%)	454 (93.8%)	403 (93.1%)
Creatinine (μmol/l)	73 (±18)	84 (±14)	62 (±13)
Diabetes	162 (17.8%)	95 (19.7%)	67 (15.7%)
Hypertension	231 (25.3%)	118 (24.4%)	113 (26.3%)
Vascular Disease (Coronary Heart Disease and Stroke)	38 (4.3%)	25 (5.2%)	13 (3.0%)
Modified Cockcroft-Gault eCCl (ml/min/1.73 m^2^)	98.8 (±23.4)	89.6 (±18.2)	108.7 (±24.4)
MDRD eGFR (ml/min/1.73 m^2^)	91.2 (±20.5)	87.4 (±17.9)	95.4 (±22.3)
CKD-EPI eGFR (ml/min/1.73 m^2^)	96.3 (±15.6)	92.4 (±14.7)	100.6 (±15.4)
DXA based Rule formula eCCl (ml/min/1.73 m^2^)	103.2 (±20.4)	96.1 (±17.1)	111.1 (±21.0)
**Number (%)**			
Modified CG – CKD 3-5	61 (6.7%)	44 (9.1%)	17 (3.9%)
MDRD – CKD 3-5	39 (4.4%)	25 (5.5%)	14 (3.3%)
CKD-EPI – CKD 3-5	18 (2.0%)	11 (2.3%)	7 (1.6%)
DXA based Rule formula – CKD3-5	5 (0.6%)	2 (0.4%)	3 (0.7%)

In the total population the estimated prevalence of CKD stages 3-5 showed up to 10 fold variability; the highest was with mCG (6.7%) followed by MDRD (4.4%), CKD-EPI (2.0%) and finally Rule formula (0.6%). In general, prevalence was higher in men compared to women (Table 1 and Additional file 1: Figure S1).

We examined whether there was any systematic bias between the different formulae according to the level of renal function. The Bland-Altman plots (Additional file 1: Figure S3) showed that the variation in the differences between formulae gets larger as the mean level of renal function increases but in a non-specific fashion except for CKD-EPI. The CKD-EPI formula shows a biased pattern: under-estimating renal function compared to the other formulae at the high end of function.

### Association between estimated creatinine production and muscle mass

Overall, lean muscle mass explained a greater proportion of the variance in 24 h creatinine production as estimated by MDRD, though this was not the case in the gender specific analyses. Overall and in the gender-specific analyses, the strength of the relationship, as measured by the regression gradient, was strongest with mCG. The strongest correlation was seen with the Rule formula, which was unsurprising as this was itself derived against DXA measures (Additional file 1: Table S1 and Additional file 1: Figure S3).

### Formula estimated adjustment of muscle mass with age

**Figure 1:**
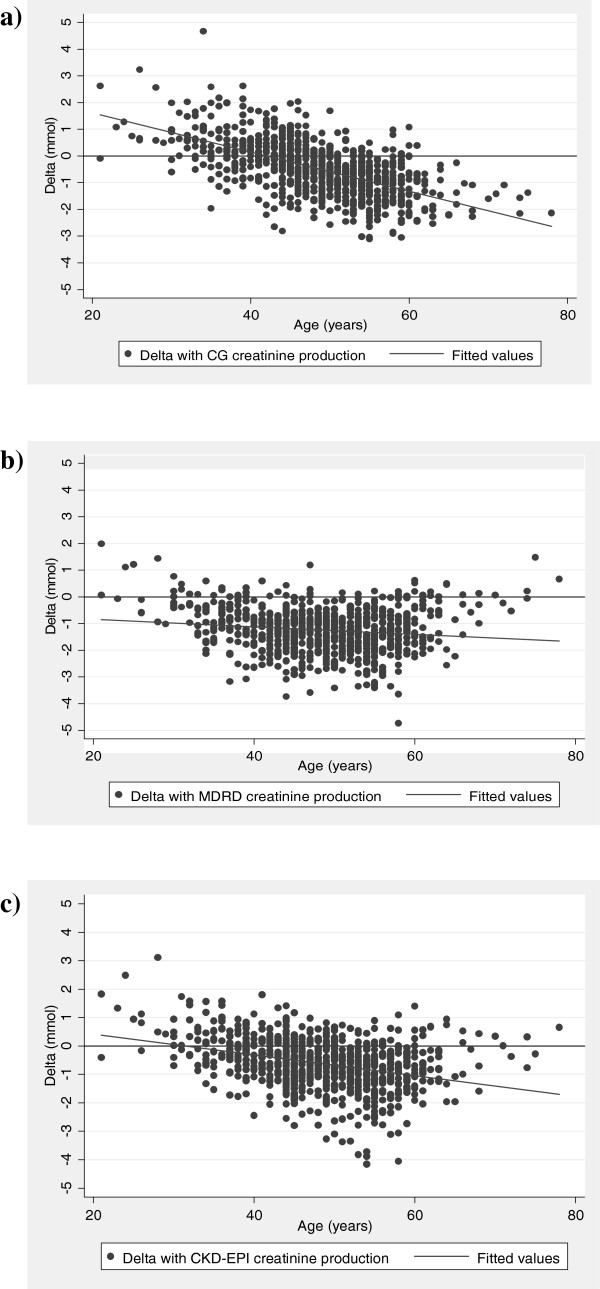
**Association between the difference in estimated 24 hour creatinine production using Cockcroft-Gault, MDRD, CKD-EPI compared to the Rule formula regressed against age of the participant. a**) Difference (delta) between 24 h creatinine production derived from Cockcroft-Gault and 24 h creatinine production from the Rule formula. **b**) Difference (delta) between 24 h creatinine production derived from MDRD and 24 h creatinine production from the Rule formula. **c**) Difference (delta) between 24 h creatinine production derived from CKD-EPI and 24 h creatinine production from the Rule formula.

As the Rule formula was most closely related to measured muscle mass it was used as the closest estimate of 24 h creatinine production. The difference between 24 h creatinine production as estimated by the other formulae and that by Rule was regressed against the subjects’ age. All formulae underestimated the 24 h creatinine production when compared to the Rule formula. Both mCG and CKD-EPI showed a marked negative association with age (regression coefficients per year increase –0.072 (95% CI –0.080 to –0.065) and –0.035 (95% CI –0.044 to –0.027) respectively so that they underestimate 24 h Cr production more in the old than the young (Figure 1). This was less marked for the MDRD formula (regression coefficients per year increase –07.012 (95% CI –0.021 to –0.003) (formal heterogeneity test between coefficients p<0.001).

### Association with vascular disease and hypertension

All formulae showed an inverse association with combined cardiac and cerebrovascular disease so that disease status was associated with worse renal function (Table 2). This was, in general, stronger for men than women. After adjustment for age these associations were attenuated; CKD-EPI had the strongest inverse association but the wide 95% confidence intervals show there is no strong evidence that one formula is superior to the others. For hypertension there was an inverse association in the crude analysis with a stronger association for women. The CKD-EPI formula had the strongest association but again overlapping 95% confidence intervals. After age adjustment the association attenuated so that they were consistent with chance. Differences in the effect estimates by gender were consistent with chance (all p-values for interaction >0.05).

**Table 2:** Odds ratios for cross-sectional associations between vascular disease (CHD and stroke) and hypertension with different estimated renal function formulae adjusted for age and stratified by gender

	**Odds Ratio – unadjusted**			**Odds Ratio – age adjusted**		
	**Total**	**Male**	**Female**	**Total**	**Male**	**Female**
**Vascular Disease (Coronary Heart Disease or Stroke)**						
Modified Cockcroft-Gault*	0.13	0.08	0.19	0.35	0.27	0.41
	(0.05-0.36)	(0.02-0.28)	(0.04-0.91)	(0.10-1.23)	(0.05-1.36)	(0.06-2.88)
	p<0.001	p<0.001	p=0.037	p=0.103	p=0.111	p=0.370
MDRD*	0.13	0.07	0.22	0.22	0.11	0.38
	(0.05-0.37)	(0.02-0.30)	(0.05-0.98)	(0.08-0.60)	(0.02-0.48)	(0.08-1.68)
	p<0.001	p<0.001	p=0.047	p=0.003	p=0.003	p=0.197
CKD-EPI*	0.09	0.05	0.15	0.17	0.09	0.28
	(0.03-0.26)	(0.01-0.18)	(0.03-0.68)	(0.06-0.51)	(0.02-0.39)	(0.06-1.35)
	p<0.001	p<0.001	p=0.013	p=0.002	p=0.001	p=0.112
Rule formula*	0.09	0.05	0.15	0.18	0.10	0.27
	(0.02-0.33)	(0.01-0.30)	(0.02-1.23)	(0.04-0.71)	(0.01-0.72)	(0.03-2.25)
	p<0.001	p=0.001	p=0.077	p=0.015	p=0.022	p=0.225
**Hypertension**						
Modified Cockcroft-Gault*	0.56	0.72	0.45	1.67	2.33	1.26
	(0.36-0.87)	(0.37-1.43)	(0.23-0.89)	(0.99-2.83)	(1.03-5.28)	(0.59-2.68)
	p=0.010	p=0.351	p=0.021	p=0.054	p=0.043	p=0.547
MDRD*	0.50	0.85	0.34	0.86	1.35	0.56
	(0.32-0.80)	(0.43-1.67)	(0.17-0.67)	(0.53-1.41)	(0.65-2.78)	(0.28-1.14)
	p=0.004	p=0.639	p=0.002	p=0.548	p=0.418	p=0.108
CKD-EPI*	0.27	0.50	0.13	0.76	1.38	0.36
	(0.15-0.48)	(0.23-1.11)	(0.05-0.36)	(0.40-1.44)	(0.56-3.41)	(0.13-0.99)
	p<0.001	p=0.088	p<0.001	p=0.394	p=0.487	p=0.048
Rule formula*	0.60	0.71	0.52	1.15	1.25	0.88
	(0.35-1.02)	(0.32-1.59)	(0.22-1.20)	(0.66-2.03)	(0.54-2.91)	(0.38-2.05)
	p=0.061	p=0.406	p=0.127	p=0.622	p=0.601	p=0.770

^*^All renal function estimates underwent log base 2 transformation.

## Discussion

This is a large study of renal function in a mixed urban-rural Indian population. The comparison with measured muscle mass provides an insight into the ability of demographic variables to estimate creatinine generation in Indian populations. This is important as the demographic variables used as surrogates for variability in creatinine production and excretion were developed in White populations and thus may not be generalisable to other ethnicities.

The mean eCCl derived from the Rule formula in healthy White Americans was 104±26 ml/min/1.73 m^2^ as compared to 103.2±20.4 ml/min/1.73 m^2^ in our Indian population [11]. It is interesting to note that the Indian population in this study had a lower mean total body mass and a lower BMI compared to the Rule’s population, but that muscle mass was comparable (Additional file 1: Table S2) showing that this Indian group is relatively lean. However, our population contained both rural and urban residents, and with a gradient of increasing obesity with urbanization recognized [20] these results should not be generalised to urban Indian populations.

Of the traditional GFR estimating equations, MDRD derived 24 h creatinine production was most strongly correlated with DXA measured muscle mass. MDRD was the only formula which did not have a marked differential bias with age and thus may be the most suitable at predicting age-related differences in muscle mass and creatinine production. The mCG formula generates the highest mean clearance but also gives the highest proportion of those with CKD 3-5, which at first sight appears paradoxical. This is explained by the distribution of creatinine clearance which appears to have a larger right sided tail using mCG (Additional file 1: Figure S1).

The Rule formula led to the highest estimate of GFR, suggesting that the other formulae underestimate true muscle mass in this population. Importantly, the estimate of the prevalence of CKD 3-5 varies from 0.6% to 6.7% depending on which formula is used. This highlights how comparisons of studies estimating the prevalence of CKD are limited if different formulae are used and has important implications for health service planning. We didn’t find evidence that one formula was better than the others in predicting vascular disease and hypertension.

This study is the largest comparison of different estimates of creatinine clearance in an Indian population which also has DXA derived measures of muscle mass. It does, however, have several important limitations. (1) *Lack of a gold standard*. We did not compare the calculated GFR and CCl estimates with a ‘measured gold standard’. However, the assumption that measured GFR is the ‘gold standard’ against which all estimating formulae should be compared has been called into question [21]. There’s no universally recognised gold standard method of measuring GFR and different methods often yield different measurements. Measured GFR has not been shown to be better than other markers of renal disease at predicting outcomes associated with poor renal function [22]. Determining with great accuracy ‘actual’ GFR is less a priority than calculating a person’s risk of clinical complications. (2) *CCl over-estimates GFR* GFR estimating equations have been developed to estimate GFR against measured GFR and underestimate measured CCl by approximately 15% [23]. CG was developed to estimate CCl by regressing UCCl/kg body weight onto age [24]. Direct comparison of CCl and GFR is therefore limited but in clinical practice they are used similarly. (3) *Estimating Muscle Mass from DXA scans* Wang et al’s original work, concluding that appendicular muscle mass constitutes approximately 75% of total body skeletal muscle mass [16], was on a sample of 25 White men. In further work ethnicity was not found to be a significant predictor of skeletal muscle, but of the 414 study subjects, only 0.12% were ‘Asian’ [25]. We applied Wang’s estimate to an Indian group, although lacking validation data in this population. However, any error should apply equally to all the compared formulae. (4) *Contribution of diet and metabolism* Creatinine is mainly produced by muscle breakdown, largely determined by muscle mass but affected by dietary protein and metabolic rate. The effect of dietary protein is minimised by ensuring serum creatinine is measured on a fasting blood sample [26], as in this study. Metabolism was not examined. (5) *Comorbidity measures* There may have been under-ascertainment of vascular end-points in rural areas, compared to urban which benefit from employment health facilities. This may have attenuated true associations.

This study supports previous evidence that formulae to estimate renal function based on serum creatinine and demographic characteristics are all imprecise due to the impossibility of predicting steady-state creatinine generation in an individual. The MDRD formula appears less variable and less biased by age in the prediction of creatinine generation. However there was no convincing evidence that any one measure was superior in predicting complications of CKD; this may be due to a low prevalence of complications in this relatively young, healthy population. Follow-up will show which estimate of renal function is most strongly associated with comorbidities. Further work is required to determine the most useful estimate of renal function in an Indian population. Given the large and increasing burden of diabetes and obesity in India, this will be of major future importance in planning future renal care services.

## Conclusions

Our analyses support the use of MDRD for estimating renal function in Indian populations. Further work is required to assess the predictive value of formulae for incident disease and complications of CKD.

## Competing interests

The authors declare that they have no competing interests.

## Authors’ contributions

SK SE YBS KR and HK contributed to study design. HK SK and SE managed the fieldwork, staff training and supervision of the data collection for the study. PB CT and YBS contributed to data analysis. PB CT YBS and DN contributed to data interpretation. PB wrote the first draft of the paper. CT YBS DN and HK contributed to the writing of the paper. All authors provided feedback on the manuscript and approved the submitted version.

## Pre-publication history

The pre-publication history for this paper can be accessed here:

http://www.biomedcentral.com/1471-2369/14/30/prepub

## Supplementary Material

Additional file 1: Table S1 Correlation and regression estimates between estimated 24 hour creatinine production using different formulae as predicted by lean muscle mass from DXA scan. **Table S2.** Comparison of Indian Study population and White American Study population in which the Rule formula was derived. **Figure S1.** Distribution of renal function by different estimates across Indian study population – vertical reference line eGFR 60 ml/min/1.73 m2 *(Below this = CKD 3 or worse). ***Figure S2.** Bland-Altman plots comparing difference between the GFR/CCl generated by one formula and that generated by another against the mean GFR/CCl of both formulae. The central horizontal line corresponds to the mean difference while the outer lines correspond to the 95% limits of agreement (Mean ± 2 S.D.). **Figure S3.** Linear regression of relationship between estimated 24 hour creatinine production derived from different formulae against measured lean muscle mass derived from DXA. Click here for file
