# Supplementary material for: Differences in estimation of creatinine generation between renal function estimating equations in an Indian population: cross-sectional data from the Hyderabad arm of the Indian migration study
Source: BMC Nephrol. 2013 Feb 4;14:30. doi: 10.1186/1471-2369-14-30 (PMC3599554; doi:10.1186/1471-2369-14-30)
Supplement: Additional file 1: Table S1 — Correlation and regression estimates between estimated 24 hour creatinine production using different formulae as predicted by lean muscle mass from DXA scan. Table S2. Comparison of Indian Study population and White American Study population in which the Rule formula was derived. Figure S1. Distribution of renal function by different estimates across Indian study population – vertical reference line eGFR 60 ml/min/1.73 m2 (Below this = CKD 3 or worse). Figure S2. Bland-Altman plots comparing difference between the GFR/CCl generated by one formula and that generated by another against the mean GFR/CCl of both formulae. The central horizontal line corresponds to the mean difference while the outer lines correspond to the 95% limits of agreement (Mean ± 2 S.D.). Figure S3. Linear regression of relationship between estimated 24 hour creatinine production derived from different formulae against measured lean muscle mass derived from DXA. [file 1471-2369-14-30-S1.doc]

Supplementary Table 1: Correlation and regression estimates between estimated 24 hour creatinine production using different formulae as predicted by lean muscle mass from DXA scan

| Estimating formula | R^2^ | Regression coefficient^*^ | 95% Confidence Interval |
| --- | --- | --- | --- |
| Men and women | | | |
| Cockcroft-Gault | 0.70 | 0.330 | 0.314, 0.345 |
| MDRD | 0.83 | 0.284 | 0.275, 0.292 |
| CKD-EPI | 0.79 | 0.314 | 0.302, 0.330 |
| Rule formula | 0.98 | 0.354 | 0.351, 0.357 |
| Men | | | |
| Cockcroft-Gault | 0.65 | 0.401 | 0.369, 0.434 |
| MDRD | 0.64 | 0.209 | 0.192, 0.225 |
| CKD-EPI | 0.56 | 0.248 | 0.222, 0.274 |
| Rule formula | 0.98 | 0.390 | 0.383, 0.396 |
| Women | | | |
| Cockcroft-Gault | 0.71 | 0.449 | 0.421, 0478 |
| MDRD | 0.66 | 0.199 | 0.184, 0.214 |
| CKD-EPI | 0.59 | 0.258 | 0.237, 0.278 |
| Rule formula | 0.98 | 0.377 | 0.371, 0.382 |

* All p-values <0.001

Supplementary Table 2: Comparison of Indian Study population and White American Study population in which the Rule formula was derived.

|  | Rule Study population – White Americans | Hyderabad subset of the Indian Migration Study |
| --- | --- | --- |
| Mean age (yrs) | 55 ± 20 | 49 ± 8 |
| Percentage female (%) | 51 | 47 |
| Mean Total Body Mass (kg) | 75.8 ± 16.6 | 65.4 ± 11.9 |
| Muscle mass corrected for Body Surface Area (kg/1.73m^2^) | 25 ± 5 | 24 ± 4 |
| Mean Body Surface Area (m^2^) | 1.85 ± 0.23 | 1.69 ± 0.18 |
| Mean Body Mass Index (kg/m^2^) | 26.6 ± 4.9 | 25.9 ± 4.4 |

Supplementary Figure 1: Distribution of renal function by different estimates across Indian study population – vertical reference line eGFR 60 ml/min/1.73m2 *(Below this = CKD 3 or greater)*

Men and women

1. Modified Cockcroft-Gault – eCCl b) MDRD – eGFR

c) CKD-EPI – eGFR d) Rule formula – eCCl

Men

1. mCG– eCCl b) MDRD – eGFR

c) CKD-EPI – eGFR d) Rule formula – eCCl

Women

1. mCG– eCCl b) MDRD – eGFR

c) CKD-EPI – eGFR d) Rule formula – eCCl

Supplementary Figure 2

Bland-Altman plots comparing difference between the GFR/CCl generated by one formula and that generated by another against the mean GFR/CCl of both formulae. The central horizontal line corresponds to the mean difference while the outer lines correspond to the 95% limits of agreement (Mean ± 2 S.D.)

Supplementary Figure 3: Linear regression of relationship between estimated 24 hour creatinine production derived from different formulae against measured lean muscle mass derived from DXA

Men and Women

1. Cockcroft-Gault b) MDRD

1. CKD-EPI d) Rule formula

Men

a) Cockcroft-Gault b) MDRD

c) CKD-EPI d) Rule formula

Women

a) Cockcroft-Gault b) MDRD

c) CKD-EPI d) Rule Formula
